# Dysregulation of MicroRNAs in cancer

**DOI:** 10.1186/1423-0127-19-90

**Published:** 2012-10-17

**Authors:** Pai-Sheng Chen, Jen-Liang Su, Mien-Chie Hung

**Affiliations:** 1 Department of Medical Laboratory Science and Biotechnology, National Cheng Kung University, Tainan, Taiwan; 2 The Institute of Basic Medical Sciences, National Cheng Kung University, Tainan, Taiwan; 3 Graduate Institute of Cancer Biology, China Medical University, Taichung, Taiwan; 4 Department of Biotechnology, Asia University, Taichung, Taiwan; 5 Center for Molecular Medicine, China Medical University Hospital, Taichung, Taiwan; 6 Department of Molecular and Cellular Oncology, The University of Texas MD Anderson Cancer Center, Houston, Texas, USA

**Keywords:** Cancer progression, miRNA biogenesis, miRNA dysregulation

## Abstract

MicroRNAs (miRNAs) are involved in multiple biological activities as well as disease progression including cancer. Interestingly, miRNAs could act as either tumor suppressors or oncogenes depending on the functions of their targets. Using high-throughput profiling, dysregulation of miRNAs has been widely observed in different stages of cancer, and there is mounting evidence demonstrating several misguided mechanisms that cause miRNA dysregulation. In this review, we summarize the key functions of miRNAs in cancer, especially those affecting tumor metastasis and drug resistance. Moreover, the mechanisms leading to dysregulation of miRNAs, including genomic abnormalities, DNA/histone modifications, transcriptional regulation, abnormal biogenesis, and interaction between miRNAs, are also discussed.

## Review

### Introduction

MicroRNAs (miRNAs) are small noncoding RNAs which enhance the cleavage or translational repression of specific mRNA with recognition site(s) in the 3’-untranslated region (3′UTR). The biogenesis of miRNA is controlled by two RNase-dependent processing steps that converts a long primary transcript into a mature ~20 nt miRNA. The mature miRNA are released and then loaded onto the miRNA-induced silencing complex (miRISC), which acts as a guiding strand to recognize specific mRNA targets. Since the discovery of miRNAs, several large-scale studies have compared the profiles of miRNA expression patterns between corresponding non-tumor and tumor tissues [1,2]. Dysregulation of miRNAs has been documented in different types of human cancers [1,2]. As miRNA expression is tissue-specific, the expression profile of miRNAs has been proposed as a marker to identify tumor origin [1]. Several studies have also suggested that the expression of miRNAs may even be a more reliable and better prognostic indicator than proteins or mRNAs under certain conditions [1,3,4]. For example, a five-miRNA signature profile could predict the cancer relapse and survival in NSCLC patients [3]. In addition, the expression of 25 miRNAs could classify tissues as normal pancreas, chronic pancreatitis, or pancreatic adenocarcinoma [5]. Currently, numerous cancer-specific miRNAs have been functionally identified, and the mechanisms underlying miRNA regulation are becoming more complete.

### Emerging roles of miRNAs in cancer

Let-7 is the most studied miRNA both in development and cancer. The human let-7 family comprises 12 closely related members of miRNA (let-7-a-1, a-2, a-3, b, c, d, e, f-1, f-2, g, i and miR-98). Johnson *et al*. reported that let-7 is downregulated in lung cancer and is associated with elevated RAS expression [6]. They further showed that let-7 is complementary to multiple sites in the 3′UTR of the human RAS genes, allowing let-7 to suppress the expression of K-RAS and N-RAS. The tumor suppressive roles of let-7 are further strengthened by its antagonistic roles toward the expression of multiple oncogenes including RAS, MYC, and other cell cycle regulators in a variety of human cancer tissues [6-8]. For example, let-7 directly targets other proto-oncogenes such as CDK6, cyclin D, CCND2, and CDC25A and represses cell proliferation by promoting the G1 to S transition [7]. In addition, high mobility group A2 (HMGA2), an oncogene frequently mutated in multiple types of cancers, is also hindered by let-7 [9]. There are seven let-7 binding sites in the 3′UTR of HMGA2 mRNA. Disrupting the interaction between let-7 and these binding sites reduces let-7-mediated HMGA2 downregulation and consequently enhances anchorage-independent growth of cancer cells [9,10]. Recent studies also suggest that let-7 regulates metastasis-associated genes such as *MYH9* and C-C chemokine receptor type 7 *(CCR7*) to facilitate invasion ability of cancer cells [11,12].

MiRNAs derived from miR-17-92 cluster, which contains seven homologous miRNAs, including miR-17-3p, miR-17-5p, miR-18a, miR-20a, miR-19a, miR-19b-1, and miR-92a-1, have been identified as oncogenic miRNAs. These miRNAs target multiple genes involved in proapoptotic pathways, reflecting their oncogenic activities [13,14]. The oncogenic roles of miR-17-92 cluster were reported by He *et al.* in which expression of this cluster accelerated c-Myc-induced lymphoma development and resulted in an advanced tumor in Eu-Myc transgenic mouse model of human B cell lymphoma [14]. The direct targets of miR-17-92 cluster have been identified to include Bim, PTEN, and p21 [13,15]. However, several controversial studies indicated that miR-17-92 possesses tumor suppressor activities. For instance, miR-17-92 cluster inhibits E2F1 to abolish Myc-induced cell proliferation, and miR-17-5p represses proliferation of breast cancer cells through targeting AIB1 [16,17].

MiR-21 has been shown to be overexpressed in a wide variety of cancers, including malignant human glioblastoma tumor tissues [18]. Knockdown of miR-21 induced activation of caspases and resulted in apoptosis in glioblastoma cells [19]. In addition, Papagiannakopoulos *et al*. indicated that knockdown of miR-21 activates the p53 pathway, mediates the induction of TGF-β signaling, and eventually suppresses cell growth, increases apoptosis, and induces cell cycle arrest in glioblastoma cells [20]. Downregulation of miR-21 also repressed cell growth in breast cancer cells by directly regulating PDCD4 tumor suppressor [21]. Moreover, Yao *et al*. reported a proliferation-promoting function of miR-21 in which knockdown of miR-21 suppressed proliferation of HeLa cells [22]. These studies suggest that miR-21 enables cells to gain their growth advantages.

#### The roles of miRNAs in tumor metastasis

**Figure 1:**
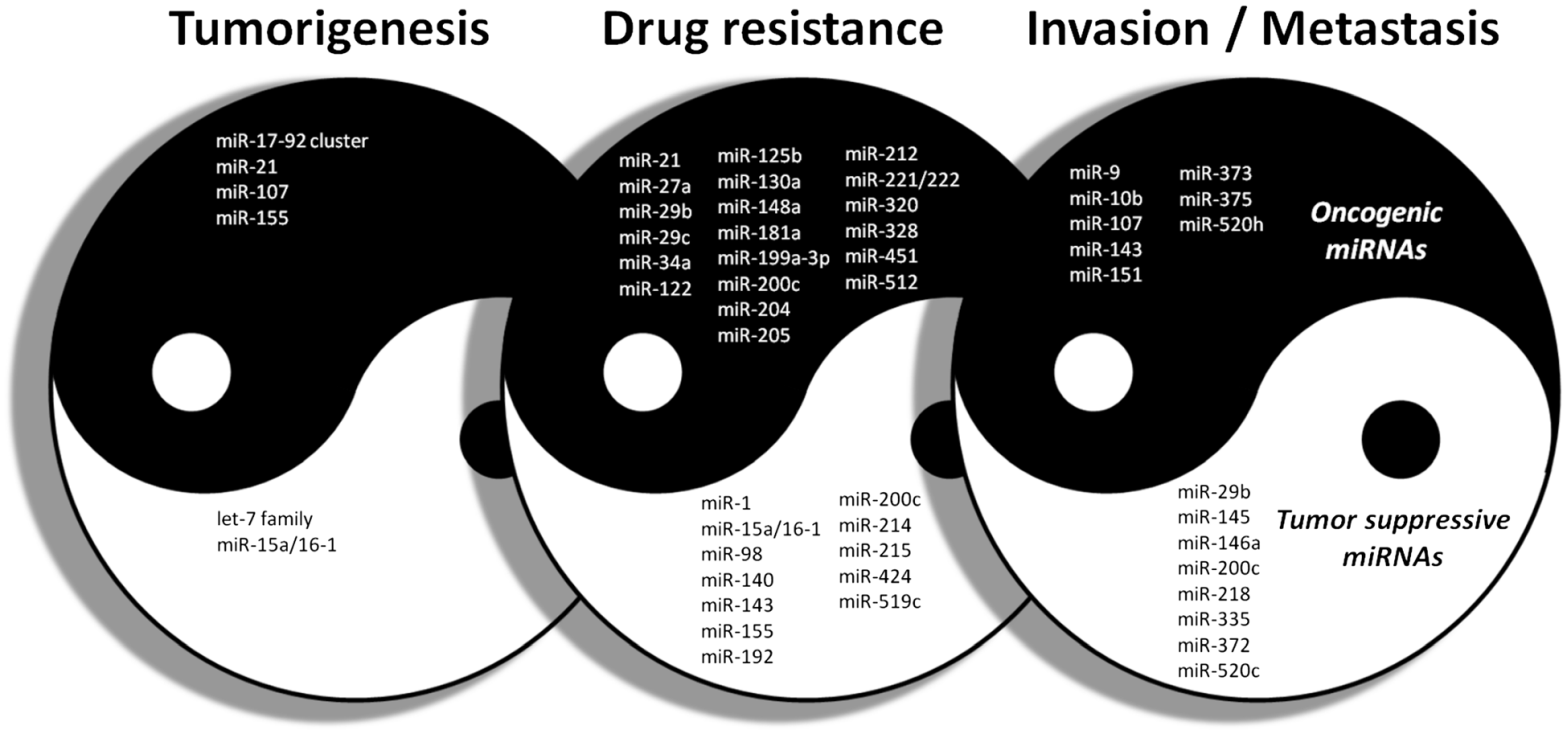
**MiRNAs functionally involved in cancer progression.** MiRNAs with characterized functions in tumorigenesis, drug resistance, and metastasis during cancer progression are summarized as either ying (oncogenic) or yang (tumor suppressive) miRNAs. See text for more detailed descriptions.

In addition to their abilities to mediate cell growth, miRNAs also affect tumor metastasis when the target genes are related to metastatic phenotypes of cancer cells (Figure 1) [23,24]. MiR-10b is the most studied miRNA with metastasis-promoting effect [25] and is directly regulated by Twist1, an oncoprotein facilitating epithelial-mesenchymal transition (EMT). Expression of miR-10b is markedly elevated and maintains the invasiveness of metastatic human breast cancer cells. Overexpression of miR-10b in non-metastatic breast cancer cells results in enhanced invasiveness and distant metastasis. MiR-10b targets *HOXD10* mRNA and enhances the expression of RhoC, a prometastatic gene suppressed by HOXD10 [25]. In addition, Tavazoie *et al*. demonstrated that SOX4 and cadherin C could be downregulated by miR-335, leading to a reduction of the metastatic ability of breast cancer cells [26]. EMT and stemness have been shown to be closely related [27]. For instance, a recently study demonstrated that miR200c is upregulated by p53, and this in turn inhibits both EMT and stemness through *ZEB1* and *BMI1*, respectively [28]. MiR-335 also acts as metastasis suppressor in neuroblastoma and gastric cancer [29,30]. CD44, an adhesion molecule that represses tumor metastasis, is suppressed by miR-373 and miR-520c [31]. Moreover, miR-373 has been identified as an oncomir in testicular germ cell tumor [32]. MiR-218 is an intronic miRNA coexpressed with its host gene, Slit, which encodes the ligand of Robo1, and downregulation of Slit reduces miR-218 expression, leading to increased Robo1 expression. As Slit interacts with Robo1 to facilitate metastasis, this pathway provides a negative feedback loop in gastric cancer [33]. Using a metastasis selection model of mouse colorectal cancer, Ding *et al*. identified a set of genes, including *APOBEC3G*, *CD133*, *LIPC*, and *S100P*, which play key roles in enhancing liver metastasis of colorectal cancer [34]. One of these genes, *APOBEC3G*, was further identified to downregulate miR-29b and subsequently restores the expression of MMP2, leading to enhanced invasion *in vitro* and metastasis *in vivo*[34]. 

#### Functions of miRNAs in drug resistance

In addition to the studies showing that miRNAs are associated with tumorigenesis and metastasis, several miRNAs have also been found to affect the drug resistance of cancer cells (Figure 1). MiR-519c was first found to increase drug sensitivity of colon cancer cells by regulating ABCG2 [35] and was later shown to suppress the expression of HIF-1α which consequently attenuates tumor angiogenesis [36]. Paradoxically, Su *et al*. showed that E1A downregulates the expression of miR-520h, induces protein phosphatase PP2A/C upregulation, suppresses IKK/NF-κB pathway, and eventually, mitigates Twist expression in breast cancer [37]. Recently, Yu *et al*. further demonstrated an oncogenic effect of miR-520h via repression of PP2A/C. Interestingly, the expression of miR-520h is inhibited by resveratrol, leading to NF-κB-mediated reduction of Forkhead box protein C2 (FOXC2) [38]. These studies indicate that miR-520 family could act as tumor suppressor or oncogenes depending on their downstream signaling. MiR-15a and miR-16-1 have been documented as tumor suppressor in chronic lymphocytic leukemia (CLL) [39]. They are clustered on human chromosome 13q14, which is frequently deleted or downregulated in CLL and some solid tumors. Because the 3′UTR region of antiapoptotic *BCL2* mRNA contains a potential binding site for these miRNAs, a deficiency in miR-15a and miR-16-1 enhances the expression of BCL2, blocking the cleavage of pro-caspase 9 and poly-ADP-ribose polymerase (PARP) required to activate the intrinsic apoptosis pathway. Further studies revealed that expression of miR-15b and miR-16 negatively regulate the Bcl-2 protein level, leading to sensitization of gastric cancer cells to anticancer drugs [40]. Another miRNA, miR-451, has been found to be downregulated in the doxorubicin-resistant breast cancer cells. While expression of miR-451 sensitized breast cancer cells to doxorubicin treatment through regulating Mdr1/P-glycoprotein [41], Zhu *et al*. identified a controversial role of miR-451 in protecting cancer cells from anticancer drugs [42]. Functional inhibition of miR-21 has been shown to dramatically reduce the topotecan-resistance of breast cancer cells [43]. The tumor suppressor function of miR-29 has also been identified in human cholangiocarcinoma [44]. Mott *et al*. observed an inverse correlation between Mcl-1 protein and miR-29b expression. They further demonstrated the ability of miR-29 to inhibit expression of Mcl-1 protein and sensitize cancer cells to TRAIL cytotoxicity through targeting a putative target site in the 3′UTR of *Mcl-1* mRNA. Later, Garzon *et al*. found that ectopic expression of miR-29b downregulates the expression of DNA methyltransferases DNMT1, DNMT3A, and DNMT3B in AML cell, resulting in increased global DNA hypomethylation and restoring the expression of tumor suppressor genes such as the CDK inhibitor p15^INK4b^ and oestrogen receptor, ESR1 [45]. In nasopharyngeal carcinoma, miR-29c also suppresses the metastasis by downregulating collagen and laminin 1 [46].

### Mechanisms of dysregulation of miRNAs in cancer

#### Genomic abnormalities

**Figure 2:**
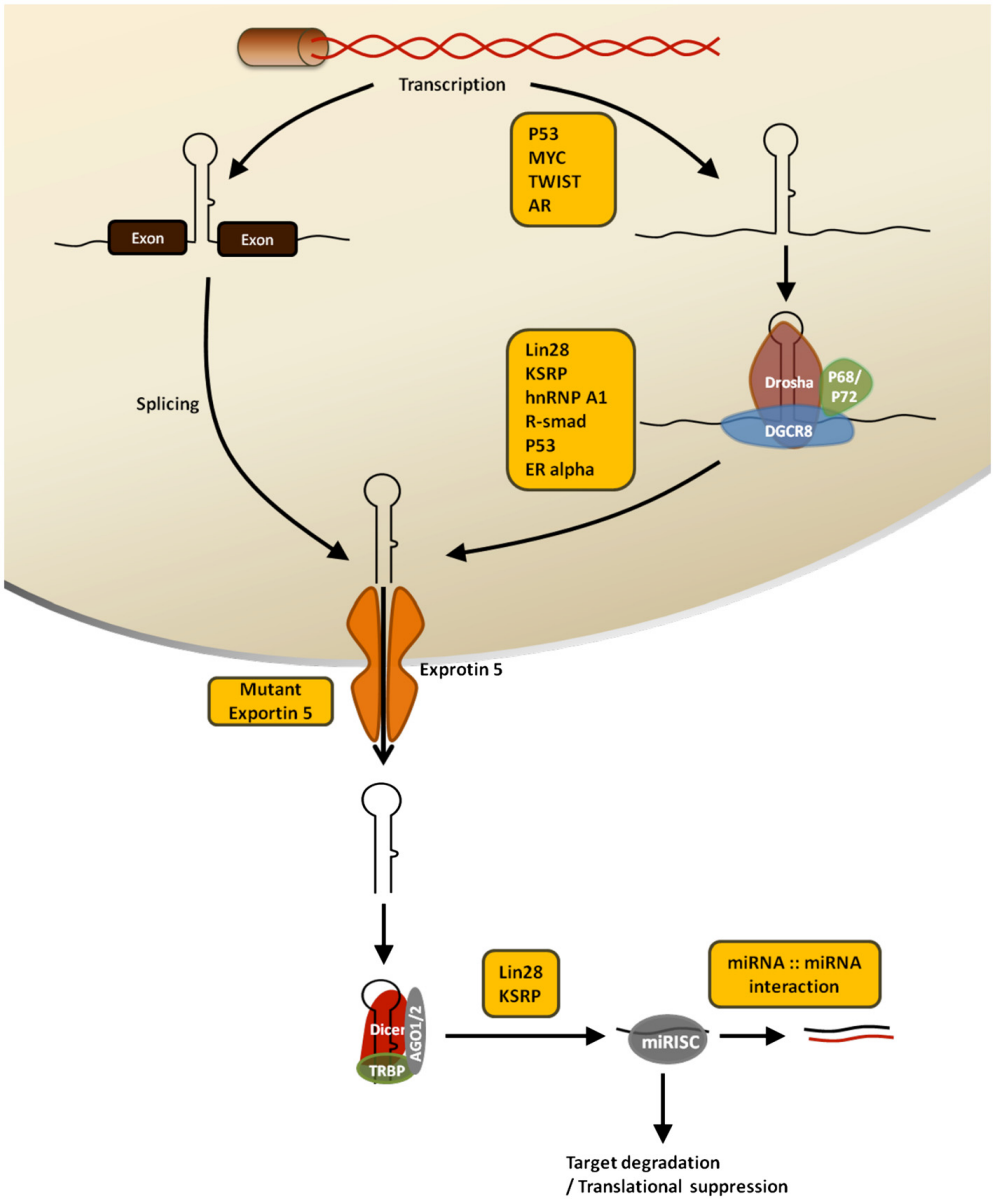
**Canonical biogenesis pathway and mechanisms of miRNA deregulation.** After RNA polymerse II-dependent transcription, pre-miRNAs are generated from pri-miRNAs or spliced RNA by Drosha-DGCR8 complex or intron splicing pathway, respectively. After exporting to cytoplasm, Dicer digests the pre-miRNAs to the mature miRNAs which guide the miRISC to inhibit target mRNAs. Factors in the yellow box indicate protein regulators or mechanisms leading to the aberrant biogenesis of miRNAs in cancer. See text for more detailed description.

Like protein-coding genes, more than half of miRNA genes in human cancers are located in chromosomal regions that frequently exhibit amplification, deletion, or translocation (Figure 2) [47]. A fundamental example of this region is 13q14 of the chromosome where *miR-15* and *miR-16* are located and frequently deleted in B cell chronic lymphocytic leukemias (B-CLL), resulting in the loss or downregulated expression of *miR-15* and *miR-16*[39,48]. In addition, using a high-throughput method, Zhang *et al.* demonstrated that deletion of *miR-17-92* cluster exists in melanomas, ovarian, and breast cancers [49]. The oncogenic miR-155 was found to be upregulated along with its host gene, *BIC,* in Burkitt's lymphoma patients [50]. These studies provide an important connection between the expression of miRNAs and genomic deletion/amplification in cancer. 

#### CpG methylation and histone modification

Transcriptional silencing of tumor suppressor genes by CpG island promoter hypermethylation is a common hallmark of cancer. Similar phenomenon has been identified in miRNA regulation in which Saito *et al*. showed that a subset of miRNAs is upregulated by treatment of inhibitors specific for DNA methylation (5-aza-2′-deoxycytidine) or histone deacetylase (4-phenylbutyric acid) in cancer cells [51]. One of these miRNAs, miR-127, is downregulated in human cancers. MiR-127 is embedded in a CpG island and dramatically upregulated through its own promoter, suggesting that DNA methylation or histone modification at this promoter region hinders the expression of miR-127 in cancer cells. The downstream target of miR-127, Bcl-6, is also consistently repressed after the treatments[51]. Lujambio *et al*. later identified another miRNA that is transcriptionally repressed in cancer cells by CpG island hypermethylation [52]. They investigated the profile of miRNA expression in cells lacking DNA methyltransferases and found that miRNA-124a is downregulated by CpG island hypermethylation. This epigenetic silencing subsequently activates CDK6 and induces Rb phosphorylation [52]. One of the let-7 genes, Let-7a-3, is also located within the CpG islands. Lu *et al*. found that let-7a-3 gene is hypermethylated in ovarian cancer and hypermethylated let-7a-3 is associated with downregulation of IGFII expression and poor prognosis in ovarian cancer patients, suggesting that *let-7* expression may target IGF-II [53]. Recently, Mazar *et al*. identified several miRNAs regulated epigenetically in melanoma. MiR-375 is one of these miRNAs with hypomethylation in melanocytes, keratinocytes, and normal skin. In contrast, tissues of melanoma exhibits hypermethylated miR-375 [54]. Overexpression of miR-375 alters the cell morphology and attenuates proliferation and invasion of melanoma cells, indicating a tumor suppressive function of miR-375 [54]. These studies explain the mechanisms of DNA/histone methylation-regulated miRNAs in human cancers.

#### Transcriptional regulation

**Figure 3:**
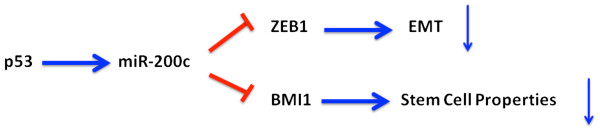
**The roles of p53-regulated miR-200c in EMT and stem-cell-like properties.** p53 directly binds to the miR-200c promoter and activates its expression. The elevated miR-200c hinders EMT via ZEB1 and reduces cell populations with stem-cell-like properties by BMI1. These pathways prevent the formation of metastatic cancer cells.

MiRNA expression is also regulated by transcription factors (Figure 2). p53 is a fundamental tumor suppressor which transcriptionally regulates hundreds of protein-coding genes. In 2007, three studies that published at the same time uncovered the subsets of miRNA regulated by p53 [55-57]. They analyzed the profiles of p53-dependent miRNA expression and found that a family of these miRNAs, miR-34a-c, was consistently upregulated by p53, which directly recognizes the promoters and activates the transcription of these miRNAs. These miRNAs function as powerful effectors to control p53-mediated cell cycle arrest and apoptosis [55-57]. As mentioned above, Chang *et al*. identified another tumor suppressor miRNA, miR-200c, that is also controlled by p53. Through binding to the miR-200c promoter, p53 induces miR-200c expression and consequently attenuates EMT and reduces stem-cell-like population in breast cancer by targeting ZEB1 and BMI1, respectively (Figure 3) [28]. Two other transcription factors, Myc and E2F1, were found to affect the expression of oncogenic miR-17-92 cluster [58,59]. These studies demonstrated that c-Myc induces expression of a miRNA cluster on human chromosome 13 by binding to this locus. E2F1, a Myc-regulated transcription factor that induces cell cycle progression, is suppressed by the miR-17-92 cluster and its paralog, miR-106b-25 [58-60]*.* As E2F1 and Myc upregulates miR-17-92, the suppressive effect on these transcription factors forms a negative feedback loop [58]. 

#### Abnormal maturation pathways

After generation of primary miRNAs, a two-step RNase-dependent maturation pathway is required to produce mature miRNAs (Figure 2). First, primary miRNAs (pri-miRNA) are processed by Drosha-containing complex to stem-loop pre-miRNAs, which are then further processed by the second RNase, Dicer, to short, double-strand duplexes. Eventually, one of the functional strands in the resulting duplexes is preserved, forming a functional complex with the RISC proteins, and acts as guiding strands for specific recognition. Currently, several RNA-binding proteins have been found to affect this canonical pathway with some that are involved in the regulation of cancer progression.

Lin-28 is the most studied RNA-binding protein being capable of regulating let-7 biogenesis. Overexpression of Lin-28 has been shown as an unfavorable prognostic marker in human cancers [61]. Lin-28 modulates the structural alternation of pre-let-7g to inhibit Dicer-dependent processing [62]. Another mechanism underlying let-7-mediated Dicer processing step also has been uncovered in which the terminal uridylyltransferase 4 (TUT4) is recruited by Lin-28 to promote uridylation of pre-let-7, and thus destabilizing pre-let-7 and blocking Dicer-dependent maturation [63]. Lin-28B, a homolog of Lin-28 (also called Lin-28A), also modulates let-7 maturation in a TUT4-independent pathway [64]. Both mechanisms result in the downregulation of mature let-7, leading to cancer progression.

The KH-type splicing regulatory protein (KSRP) was identified to enhance both Drosha- and Dicer-mediated miRNA processing through interaction with specific sequences in the loop region of a subset of pri-miRNAs [65]. Knockdown of KSRP represses the expression of specific mature miRNAs, such as let-7a and miR-206, and consequently affects cell proliferation and differentiation. Regulation at the pri-miRNA to pre-miRNA processing step is also affected by hnRNP A1, a nucleocytoplasmic shuttling heterogeneous nuclear ribonucleoprotein. HnRNP A1 facilitates pri-miR-18a for conversion into pre-miR-18a [66] and recognizes the highly-conserved loop region of miR-18a, resulting in a structural rearrangement of this hairpin to generate a more favorable cleavage site for Drosha [67]. Furthermore, upon binding to hnRNP A1, pri-let-7a-1 is unable to be processed by KSRP because the conserved binding site of hnRNP A1 for pri-let-7a overlaps with that of KSRP [68].

The two DEAD-box RNA helicases, p68 (DDX5) and p72 (DDX17), are components of the Drosha microprocessor complex [69]. Recently, protein factors associating with the Drosha-p68 or Drosha-p72 have been identified as key regulators controlling miRNA biogenesis. The transforming growth factor-β (TGF-β) family and its signal transducers, Smads, play important roles during cancer progression. TGF-β and one of its family members, the bone morphogenetic protein 4 (BMP4), was found to induce the expression of mature miR-21 through an R-smad-dependent pathway [70]. This effect was further identified to be posttranscriptional, as they upregulate pre- and mature miR-21 without affecting pri-miR-21 [70]. R-smad interacts and stabilizes Drosha-p68 complex on the pri-miR-21 hairpin, thus promoting the maturation of miR-21. In addition to its function as a transcription factor, p53 was also identified to modulate miRNA biogenesis directly by binding to Drosha-p68 complex [71]. A subset of miRNAs, such as miR-143 and miR-16, are induced posttranscriptionally under DNA damage condition, whereas this effect could not be observed in p53-null HCT116 cells [71]. Another report indicated that estrogen receptor-α (ERα) also interacts with Drosha-p72 complex, leading to a reduced affinity of Drosha complex to a subset of miRNA in the presence of estradiol [72]. Recently, Kawai *et al.* demonstrated that breast cancer 1 (BRCA1), a human tumor suppressor gene, regulates miRNA biogenesis by recognizing pri-miRNA and binding to the Drosha microprocessor and Smads/p53, which enhances processing of a subset of miRNAs [73]. As the above-mentioned protein factors are critical determinants during cancer progression, it would be interesting to investigate the detailed mechanisms mediating miRNA biogenesis in the context of cancer.

#### miRNA-miRNA interaction

After processing, mature miRNAs are produced as functional strands, loaded onto miRISC, and targeted to specific 3′UTRs, thereafter. In addition to the binding between miRNA and 3′UTR of its target mRNA, Chen *et al*. recently identified a direct interaction between two individual miRNAs, miR-107 and let-7 (Figure 2). This study provides the first evidence that two different miRNAs could interact directly with each other through sequence match [74]. Using a mutation system, Chen *et al*. further identified the essential role of an internal loop within the miR-107::let-7 duplex, which provides important clues for further investigation on the underlying mechanism [74]. MiR-107 mitigates the tumor suppressive effects of let-7, and thus facilitating cancer progression. As endogenous let-7 is capable of suppressing the expression of multiple oncogenes including Ras and Hmga2, inhibition of let-7 allows cancer cells becoming aggressive. During the progression of cancer, overexpressed miR-107 targets and destabilizes let-7, enabling oncoproteins to escape from let-7-mediated suppression. Another study simultaneously published by Tang *et al*. also provides functional evidence of miRNA-miRNA interaction between miR-709 and pri-miR-15a/16-1 [75]. This newly discovered regulation sheds light on our current knowledge in the posttranscriptional control of miRNA. Considering the interaction and the multi-faceted roles of a given miRNA may have, the regulation network of miRNA becomes more complex than we originally thought.

## Conclusion

MiRNAs have been known to function in most physiological processes in humans. As dysregulated expression of specific miRNAs is a common phenomenon observed in human cancers, unraveling the underlying mechanisms misguided at each step of miRNA biogenesis is crucial to our knowledge on how these miRNAs are altered. Accumulating evidence in both transcriptional and posttranscriptional regulation have enabled us to understand the novel functions of classical transcription factors, and more interestingly, RNA binding proteins, in controlling cancer-specific miRNAs. Because miRNAs play key roles in human cancer, identifying the underlying pathways will provide a more complete understanding of their functions and regulations during cancer progression and may have clinical applications in the future.

## Competing interests

The authors have no conflicts of interest to declare.

## Authors’ contributions

P-SC, J-LS, and M-CH equally conceived and prepared this review. All of the authors read and approved the final manuscript.

## References

[B1] Lu J Getz G Miska EA Alvarez-Saavedra E Lamb J Peck D Sweet-Cordero A Ebert BL Mak RH Ferrando AA Downing JR Jacks T Horvitz HR Golub TR MicroRNA expression profiles classify human cancers Nature 2005 435 7043 834 838 10.1038/nature03702 15944708

[B2] Calin GA Croce CM MicroRNA signatures in human cancers Nat Rev Cancer 2006 6 11 857 866 10.1038/nrc1997 17060945

[B3] Yu SL Chen HY Chang GC Chen CY Chen HW Singh S Cheng CL Yu CJ Lee YC Chen HS Su TJ Chiang CC Li HN Hong QS Su HY Chen CC Chen WJ Liu CC Chan WK Li KC Chen JJ Yang PC MicroRNA signature predicts survival and relapse in lung cancer Cancer Cell 2008 13 1 48 57 10.1016/j.ccr.2007.12.008 18167339

[B4] Calin GA Ferracin M Cimmino A Di Leva G Shimizu M Wojcik SE Iorio MV Visone R Sever NI Fabbri M Iuliano R Palumbo T Pichiorri F Roldo C Garzon R Sevignani C Rassenti L Alder H Volinia S Liu CG Kipps TJ Negrini M Croce CM A MicroRNA signature associated with prognosis and progression in chronic lymphocytic leukemia N Engl J Med 2005 353 17 1793 1801 10.1056/NEJMoa050995 16251535

[B5] Bloomston M Frankel WL Petrocca F Volinia S Alder H Hagan JP Liu CG Bhatt D Taccioli C Croce CM MicroRNA expression patterns to differentiate pancreatic adenocarcinoma from normal pancreas and chronic pancreatitis Jama 2007 297 17 1901 1908 10.1001/jama.297.17.1901 17473300

[B6] Johnson SM Grosshans H Shingara J Byrom M Jarvis R Cheng A Labourier E Reinert KL Brown D Slack FJ RAS is regulated by the let-7 microRNA family Cell 2005 120 5 635 647 10.1016/j.cell.2005.01.014 15766527

[B7] Kumar MS Erkeland SJ Pester RE Chen CY Ebert MS Sharp PA Jacks T Suppression of non-small cell lung tumor development by the let-7 microRNA family Proc Natl Acad Sci U S A 2008 105 10 3903 3908 10.1073/pnas.0712321105 18308936 PMC2268826

[B8] Nadiminty N Tummala R Lou W Zhu Y Shi XB Zou JX Chen H Zhang J Chen X Luo J Devere White RW Kung HJ Evans CP Gao AC MicroRNA let-7c Is Downregulated in Prostate Cancer and Suppresses Prostate Cancer Growth PLoS One 2012 7 3 e32832 10.1371/journal.pone.0032832 22479342 PMC3316551

[B9] Mayr C Hemann MT Bartel DP Disrupting the pairing between let-7 and Hmga2 enhances oncogenic transformation Science 2007 315 5818 1576 1579 10.1126/science.1137999 17322030 PMC2556962

[B10] Lee YS Dutta A The tumor suppressor microRNA let-7 represses the HMGA2 oncogene Genes Dev 2007 21 9 1025 1030 10.1101/gad.1540407 17437991 PMC1855228

[B11] Kim SJ Shin JY Lee KD Bae YK Sung KW Nam SJ Chun KH MicroRNA let-7a suppresses breast cancer cell migration and invasion through downregulation of C-C chemokine receptor type 7 Breast Cancer Res 2012 14 1 R14 10.1186/bcr3098 22251626 PMC3496131

[B12] Liang S He L Zhao X Miao Y Gu Y Guo C Xue Z Dou W Hu F Wu K Nie Y Fan D MicroRNA let-7f inhibits tumor invasion and metastasis by targeting MYH9 in human gastric cancer PLoS One 2011 6 4 e18409 10.1371/journal.pone.0018409 21533124 PMC3078939

[B13] Olive V Bennett MJ Walker JC Ma C Jiang I Cordon-Cardo C Li QJ Lowe SW Hannon GJ He L miR-19 is a key oncogenic component of mir-17-92 Genes Dev 2009 23 24 2839 2849 10.1101/gad.1861409 20008935 PMC2800084

[B14] He L Thomson JM Hemann MT Hernando-Monge E Mu D Goodson S Powers S Cordon-Cardo C Lowe SW Hannon GJ Hammond SM A microRNA polycistron as a potential human oncogene Nature 2005 435 7043 828 833 10.1038/nature03552 15944707 PMC4599349

[B15] Hong L Lai M Chen M Xie C Liao R Kang YJ Xiao C Hu WY Han J Sun P The miR-17-92 cluster of microRNAs confers tumorigenicity by inhibiting oncogene-induced senescence Cancer Res 2010 70 21 8547 8557 10.1158/0008-5472.CAN-10-1938 20851997 PMC2970743

[B16] Coller HA Forman JJ Legesse-Miller A "Myc'ed messages": myc induces transcription of E2F1 while inhibiting its translation via a microRNA polycistron PLoS Genet 2007 3 8 e146 10.1371/journal.pgen.0030146 17784791 PMC1959363

[B17] Hossain A Kuo MT Saunders GF Mir-17-5p regulates breast cancer cell proliferation by inhibiting translation of AIB1 mRNA Mol Cell Biol 2006 26 21 8191 8201 10.1128/MCB.00242-06 16940181 PMC1636750

[B18] Chan JA Krichevsky AM Kosik KS MicroRNA-21 is an antiapoptotic factor in human glioblastoma cells Cancer Res 2005 65 14 6029 6033 10.1158/0008-5472.CAN-05-0137 16024602

[B19] Corsten MF Miranda R Kasmieh R Krichevsky AM Weissleder R Shah K MicroRNA-21 knockdown disrupts glioma growth in vivo and displays synergistic cytotoxicity with neural precursor cell delivered S-TRAIL in human gliomas Cancer Res 2007 67 19 8994 9000 10.1158/0008-5472.CAN-07-1045 17908999

[B20] Papagiannakopoulos T Shapiro A Kosik KS MicroRNA-21 targets a network of key tumor-suppressive pathways in glioblastoma cells Cancer Res 2008 68 19 8164 8172 10.1158/0008-5472.CAN-08-1305 18829576

[B21] Frankel LB Christoffersen NR Jacobsen A Lindow M Krogh A Lund AH Programmed cell death 4 (PDCD4) is an important functional target of the microRNA miR-21 in breast cancer cells J Biol Chem 2008 283 2 1026 1033 10.1074/jbc.M707224200 17991735

[B22] Yao Q Xu H Zhang QQ Zhou H Qu LH MicroRNA-21 promotes cell proliferation and down-regulates the expression of programmed cell death 4 (PDCD4) in HeLa cervical carcinoma cells Biochem Biophys Res Commun 2009 388 3 539 542 10.1016/j.bbrc.2009.08.044 19682430

[B23] Ma L Weinberg RA Micromanagers of malignancy: role of microRNAs in regulating metastasis Trends Genet 2008 24 9 448 456 10.1016/j.tig.2008.06.004 18674843

[B24] Nicoloso MS Spizzo R Shimizu M Rossi S Calin GA MicroRNAs–the micro steering wheel of tumour metastases Nat Rev Cancer 2009 9 4 293 302 10.1038/nrc2619 19262572

[B25] Ma L Teruya-Feldstein J Weinberg RA Tumour invasion and metastasis initiated by microRNA-10b in breast cancer Nature 2007 449 7163 682 688 10.1038/nature06174 17898713

[B26] Tavazoie SF Alarcon C Oskarsson T Padua D Wang Q Bos PD Gerald WL Massague J Endogenous human microRNAs that suppress breast cancer metastasis Nature 2008 451 7175 147 152 10.1038/nature06487 18185580 PMC2782491

[B27] Mani SA Guo W Liao MJ Eaton EN Ayyanan A Zhou AY Brooks M Reinhard F Zhang CC Shipitsin M Campbell LL Polyak K Brisken C Yang J Weinberg RA The epithelial-mesenchymal transition generates cells with properties of stem cells Cell 2008 133 4 704 715 10.1016/j.cell.2008.03.027 18485877 PMC2728032

[B28] Chang CJ Chao CH Xia W Yang JY Xiong Y Li CW Yu WH Rehman SK Hsu JL Lee HH Liu M Chen CT Yu D Hung MC p53 regulates epithelial-mesenchymal transition and stem cell properties through modulating miRNAs Nat Cell Biol 2011 13 3 317 323 10.1038/ncb2173 21336307 PMC3075845

[B29] Xu Y MicroRNA-335 acts as a metastasis suppressor in gastric cancer by targeting Bcl-w and specificity protein 1 Oncogene 2011 31 11 1398 407 21822301 10.1038/onc.2011.340 PMC3312408

[B30] Lynch J MiRNA-335 Suppresses Neuroblastoma Cell Invasiveness By Direct Targeting of Multiple Genes from the non-Canonical TGF-beta Signalling Pathway Carcinogenesis 2012 33 5 976 85 10.1093/carcin/bgs114 22382496 PMC3334516

[B31] Huang Q Gumireddy K Schrier M le Sage C Nagel R Nair S Egan DA Li A Huang G Klein-Szanto AJ Gimotty PA Katsaros D Coukos G Zhang L Pure E Agami R The microRNAs miR-373 and miR-520c promote tumour invasion and metastasis Nat Cell Biol 2008 10 2 202 210 10.1038/ncb1681 18193036

[B32] Voorhoeve PM le Sage C Schrier M Gillis AJ Stoop H Nagel R Liu YP van Duijse J Drost J Griekspoor A Zlotorynski E Yabuta N De Vita G Nojima H Looijenga LH Agami R A genetic screen implicates miRNA-372 and miRNA-373 as oncogenes in testicular germ cell tumors Cell 2006 124 6 1169 1181 10.1016/j.cell.2006.02.037 16564011

[B33] Tie J Pan Y Zhao L Wu K Liu J Sun S Guo X Wang B Gang Y Zhang Y Li Q Qiao T Zhao Q Nie Y Fan D MiR-218 inhibits invasion and metastasis of gastric cancer by targeting the Robo1 receptor PLoS Genet 2010 6 3 e1000879 10.1371/journal.pgen.1000879 20300657 PMC2837402

[B34] Ding Q Chang CJ Xie X Xia W Yang JY Wang SC Wang Y Xia J Chen L Cai C Li H Yen CJ Kuo HP Lee DF Lang J Huo L Cheng X Chen YJ Li CW Jeng LB Hsu JL Li LY Tan A Curley SA Ellis LM Dubois RN Hung MC APOBEC3G promotes liver metastasis in an orthotopic mouse model of colorectal cancer and predicts human hepatic metastasis The Journal of clinical investigation 2011 121 11 4526 4536 10.1172/JCI45008 21985787 PMC3204827

[B35] To KK Robey RW Knutsen T Zhan Z Ried T Bates SE Escape from hsa-miR-519c enables drug-resistant cells to maintain high expression of ABCG2 Mol Cancer Ther 2009 8 10 2959 2968 10.1158/1535-7163.MCT-09-0292 19825807 PMC3680354

[B36] Cha ST Chen PS Johansson G Chu CY Wang MY Jeng YM Yu SL Chen JS Chang KJ Jee SH Tan CT Lin MT Kuo ML MicroRNA-519c suppresses hypoxia-inducible factor-1alpha expression and tumor angiogenesis Cancer Res 2010 70 7 2675 2685 10.1158/0008-5472.CAN-09-2448 20233879

[B37] Su JL Chen PB Chen YH Chen SC Chang YW Jan YH Cheng X Hsiao M Hung MC Downregulation of microRNA miR-520h by E1A contributes to anticancer activity Cancer Res 2010 70 12 5096 5108 10.1158/0008-5472.CAN-09-4148 20501832 PMC2891368

[B38] Yu YH MiR-520h-mediated FOXC2 regulation is critical for inhibition of lung cancer progression by resveratrol Oncogene 2012 [Epub ahead of print] 10.1038/onc.2012.74 22410781

[B39] Cimmino A Calin GA Fabbri M Iorio MV Ferracin M Shimizu M Wojcik SE Aqeilan RI Zupo S Dono M Rassenti L Alder H Volinia S Liu CG Kipps TJ Negrini M Croce CM miR-15 and miR-16 induce apoptosis by targeting BCL2 Proc Natl Acad Sci U S A 2005 102 39 13944 13949 10.1073/pnas.0506654102 16166262 PMC1236577

[B40] Xia L Zhang D Du R Pan Y Zhao L Sun S Hong L Liu J Fan D miR-15b and miR-16 modulate multidrug resistance by targeting BCL2 in human gastric cancer cells Int J Cancer 2008 123 2 372 379 10.1002/ijc.23501 18449891

[B41] Kovalchuk O Filkowski J Meservy J Ilnytskyy Y Tryndyak VP Chekhun VF Pogribny IP Involvement of microRNA-451 in resistance of the MCF-7 breast cancer cells to chemotherapeutic drug doxorubicin Mol Cancer Ther 2008 7 7 2152 2159 10.1158/1535-7163.MCT-08-0021 18645025

[B42] Zhu H Wu H Liu X Evans BR Medina DJ Liu CG Yang JM Role of MicroRNA miR-27a and miR-451 in the regulation of MDR1/P-glycoprotein expression in human cancer cells Biochem Pharmacol 2008 76 5 582 588 10.1016/j.bcp.2008.06.007 18619946 PMC2628586

[B43] Si ML Zhu S Wu H Lu Z Wu F Mo YY miR-21-mediated tumor growth Oncogene 2007 26 19 2799 2803 10.1038/sj.onc.1210083 17072344

[B44] Mott JL Kobayashi S Bronk SF Gores GJ mir-29 regulates Mcl-1 protein expression and apoptosis Oncogene 2007 26 42 6133 6140 10.1038/sj.onc.1210436 17404574 PMC2432524

[B45] Garzon R Liu S Fabbri M Liu Z Heaphy CE Callegari E Schwind S Pang J Yu J Muthusamy N Havelange V Volinia S Blum W Rush LJ Perrotti D Andreeff M Bloomfield CD Byrd JC Chan K Wu LC Croce CM Marcucci G MicroRNA-29b induces global DNA hypomethylation and tumor suppressor gene reexpression in acute myeloid leukemia by targeting directly DNMT3A and 3B and indirectly DNMT1 Blood 2009 113 25 6411 6418 10.1182/blood-2008-07-170589 19211935 PMC2710934

[B46] Sengupta S den Boon JA Chen IH Newton MA Stanhope SA Cheng YJ Chen CJ Hildesheim A Sugden B Ahlquist P MicroRNA 29c is down-regulated in nasopharyngeal carcinomas, up-regulating mRNAs encoding extracellular matrix proteins Proc Natl Acad Sci U S A 2008 105 15 5874 5878 10.1073/pnas.0801130105 18390668 PMC2311339

[B47] Calin GA Sevignani C Dumitru CD Hyslop T Noch E Yendamuri S Shimizu M Rattan S Bullrich F Negrini M Croce CM Human microRNA genes are frequently located at fragile sites and genomic regions involved in cancers Proc Natl Acad Sci U S A 2004 101 9 2999 3004 10.1073/pnas.0307323101 14973191 PMC365734

[B48] Calin GA Dumitru CD Shimizu M Bichi R Zupo S Noch E Aldler H Rattan S Keating M Rai K Rassenti L Kipps T Negrini M Bullrich F Croce CM Frequent deletions and down-regulation of micro- RNA genes miR15 and miR16 at 13q14 in chronic lymphocytic leukemia Proc Natl Acad Sci U S A 2002 99 24 15524 15529 10.1073/pnas.242606799 12434020 PMC137750

[B49] Zhang L Huang J Yang N Greshock J Megraw MS Giannakakis A Liang S Naylor TL Barchetti A Ward MR Yao G Medina A O'Brien-Jenkins A Katsaros D Hatzigeorgiou A Gimotty PA Weber BL Coukos G microRNAs exhibit high frequency genomic alterations in human cancer Proc Natl Acad Sci U S A 2006 103 24 9136 9141 10.1073/pnas.0508889103 16754881 PMC1474008

[B50] Metzler M Wilda M Busch K Viehmann S Borkhardt A High expression of precursor microRNA-155/BIC RNA in children with Burkitt lymphoma Genes Chromosomes Cancer 2004 39 2 167 169 10.1002/gcc.10316 14695998

[B51] Saito Y Liang G Egger G Friedman JM Chuang JC Coetzee GA Jones PA Specific activation of microRNA-127 with downregulation of the proto-oncogene BCL6 by chromatin-modifying drugs in human cancer cells Cancer Cell 2006 9 6 435 443 10.1016/j.ccr.2006.04.020 16766263

[B52] Lujambio A Ropero S Ballestar E Fraga MF Cerrato C Setien F Casado S Suarez-Gauthier A Sanchez-Cespedes M Git A Spiteri I Das PP Caldas C Miska E Esteller M Genetic unmasking of an epigenetically silenced microRNA in human cancer cells Cancer Res 2007 67 4 1424 1429 10.1158/0008-5472.CAN-06-4218 17308079

[B53] Lu L Katsaros D de la Longrais IA Sochirca O Yu H Hypermethylation of let-7a-3 in epithelial ovarian cancer is associated with low insulin-like growth factor-II expression and favorable prognosis Cancer Res 2007 67 21 10117 10122 10.1158/0008-5472.CAN-07-2544 17974952

[B54] Mazar J DeBlasio D Govindarajan SS Zhang S Perera RJ Epigenetic regulation of microRNA-375 and its role in melanoma development in humans FEBS Lett 2011 585 15 2467 2476 10.1016/j.febslet.2011.06.025 21723283

[B55] He L He X Lim LP de Stanchina E Xuan Z Liang Y Xue W Zender L Magnus J Ridzon D Jackson AL Linsley PS Chen C Lowe SW Cleary MA Hannon GJ A microRNA component of the p53 tumour suppressor network Nature 2007 447 7148 1130 1134 10.1038/nature05939 17554337 PMC4590999

[B56] Raver-Shapira N Marciano E Meiri E Spector Y Rosenfeld N Moskovits N Bentwich Z Oren M Transcriptional activation of miR-34a contributes to p53-mediated apoptosis Mol Cell 2007 26 5 731 743 10.1016/j.molcel.2007.05.017 17540598

[B57] Chang TC Wentzel EA Kent OA Ramachandran K Mullendore M Lee KH Feldmann G Yamakuchi M Ferlito M Lowenstein CJ Arking DE Beer MA Maitra A Mendell JT Transactivation of miR-34a by p53 broadly influences gene expression and promotes apoptosis Mol Cell 2007 26 5 745 752 10.1016/j.molcel.2007.05.010 17540599 PMC1939978

[B58] O'Donnell KA Wentzel EA Zeller KI Dang CV Mendell JT c-Myc-regulated microRNAs modulate E2F1 expression Nature 2005 435 7043 839 843 10.1038/nature03677 15944709

[B59] Chang TC Yu D Lee YS Wentzel EA Arking DE West KM Dang CV Thomas-Tikhonenko A Mendell JT Widespread microRNA repression by Myc contributes to tumorigenesis Nat Genet 2008 40 1 43 50 10.1038/ng.2007.30 18066065 PMC2628762

[B60] Petrocca F Visone R Onelli MR Shah MH Nicoloso MS de Martino I Iliopoulos D Pilozzi E Liu CG Negrini M Cavazzini L Volinia S Alder H Ruco LP Baldassarre G Croce CM Vecchione A E2F1-regulated microRNAs impair TGFbeta-dependent cell-cycle arrest and apoptosis in gastric cancer Cancer Cell 2008 13 3 272 286 10.1016/j.ccr.2008.02.013 18328430

[B61] Viswanathan SR Powers JT Einhorn W Hoshida Y Ng TL Toffanin S O'Sullivan M Lu J Phillips LA Lockhart VL Shah SP Tanwar PS Mermel CH Beroukhim R Azam M Teixeira J Meyerson M Hughes TP Llovet JM Radich J Mullighan CG Golub TR Sorensen PH Daley GQ Lin28 promotes transformation and is associated with advanced human malignancies Nat Genet 2009 41 7 843 848 10.1038/ng.392 19483683 PMC2757943

[B62] Lightfoot HL Bugaut A Armisen J Lehrbach NJ Miska EA Balasubramanian S A LIN28-dependent structural change in pre-let-7g directly inhibits dicer processing Biochemistry 2011 50 35 7514 7521 10.1021/bi200851d 21815640 PMC3361669

[B63] Hagan JP Piskounova E Gregory RI Lin28 recruits the TUTase Zcchc11 to inhibit let-7 maturation in mouse embryonic stem cells Nat Struct Mol Biol 2009 16 10 1021 1025 10.1038/nsmb.1676 19713958 PMC2758923

[B64] Piskounova E Polytarchou C Thornton JE LaPierre RJ Pothoulakis C Hagan JP Iliopoulos D Gregory RI Lin28A and Lin28B inhibit let-7 microRNA biogenesis by distinct mechanisms Cell 2011 147 5 1066 1079 10.1016/j.cell.2011.10.039 22118463 PMC3227872

[B65] Trabucchi M Briata P Garcia-Mayoral M Haase AD Filipowicz W Ramos A Gherzi R Rosenfeld MG The RNA-binding protein KSRP promotes the biogenesis of a subset of microRNAs Nature 2009 459 7249 1010 1014 10.1038/nature08025 19458619 PMC2768332

[B66] Guil S Caceres JF The multifunctional RNA-binding protein hnRNP A1 is required for processing of miR-18a Nat Struct Mol Biol 2007 14 7 591 596 10.1038/nsmb1250 17558416

[B67] Michlewski G Guil S Semple CA Caceres JF Posttranscriptional regulation of miRNAs harboring conserved terminal loops Mol Cell 2008 32 3 383 393 10.1016/j.molcel.2008.10.013 18995836 PMC2631628

[B68] Michlewski G Caceres JF Antagonistic role of hnRNP A1 and KSRP in the regulation of let-7a biogenesis Nat Struct Mol Biol 2010 17 8 1011 1018 10.1038/nsmb.1874 20639884 PMC2923024

[B69] Shiohama A Sasaki T Noda S Minoshima S Shimizu N Nucleolar localization of DGCR8 and identification of eleven DGCR8-associated proteins Exp Cell Res 2007 313 20 4196 4207 10.1016/j.yexcr.2007.07.020 17765891

[B70] Davis BN Hilyard AC Lagna G Hata A SMAD proteins control DROSHA-mediated microRNA maturation Nature 2008 454 7200 56 61 10.1038/nature07086 18548003 PMC2653422

[B71] Suzuki HI Yamagata K Sugimoto K Iwamoto T Kato S Miyazono K Modulation of microRNA processing by p53 Nature 2009 460 7254 529 533 10.1038/nature08199 19626115

[B72] Yamagata K Fujiyama S Ito S Ueda T Murata T Naitou M Takeyama K Minami Y O'Malley BW Kato S Maturation of microRNA is hormonally regulated by a nuclear receptor Mol Cell 2009 36 2 340 347 10.1016/j.molcel.2009.08.017 19854141

[B73] Kawai S Amano A BRCA1 regulates microRNA biogenesis via the DROSHA microprocessor complex J Cell Biol 2012 197 2 201 208 10.1083/jcb.201110008 22492723 PMC3328391

[B74] Chen PS Su JL Cha ST Tarn WY Wang MY Hsu HC Lin MT Chu CY Hua KT Chen CN Kuo TC Chang KJ Hsiao M Chang YW Chen JS Yang PC Kuo ML miR-107 promotes tumor progression by targeting the let-7 microRNA in mice and humans J Clin Invest 2011 121 9 3442 3455 10.1172/JCI45390 21841313 PMC3163949

[B75] Tang R Li L Zhu D Hou D Cao T Gu H Zhang J Chen J Zhang CY Zen K Mouse miRNA-709 directly regulates miRNA-15a/16-1 biogenesis at the posttranscriptional level in the nucleus: evidence for a microRNA hierarchy system Cell Res 2012 22 3 504 515 10.1038/cr.2011.137 21862971 PMC3292299

